# Combined Strength of Standardized Lab Sprint Testing and Wheelchair Mobility Field Testing in Wheelchair Tennis Players

**DOI:** 10.1097/PHM.0000000000002717

**Published:** 2025-02-24

**Authors:** Rowie J.F. Janssen, Marit P. van Dijk, Thomas Rietveld, Sonja de Groot, Lucas H.V. van der Woude, Han Houdijk, Riemer J.K. Vegter

**Affiliations:** From the 1 University of Groningen, University Medical Center Groningen, Center for Human Movement Sciences, the Netherlands; 2 Department of Biomechanical Engineering, Delft University of Technology, Delft, the Netherlands; 3 Peter Harrison Centre for Disability Sports, School of Sport, Exercise and Health Sciences, Loughborough University, Loughborough, United Kingdom; 4 Department of Human Movement Sciences, Faculty of Behavioural and Movement Sciences, VU University, Amsterdam, the Netherlands; 5 Amsterdam Rehabilitation Research Center Reade, Amsterdam, The Netherlands; 6 Center for Rehabilitation, University Medical Center Groningen, Groningen, the Netherlands.

**Keywords:** Para-athletes, Adaptive Sports, Ergometry, Inertial Measurement Units, Biomechanics

## Abstract

**Objective:**

This cross-sectional study examined associations between wheelchair sprint and anaerobic power (measured in the lab) and wheelchair mobility performance (measured in the field) among two groups of wheelchair tennis players. Additionally, construct validity was assessed for both lab and field tests.

**Design:**

Nine amateur and nine elite wheelchair tennis players performed a Sprint and Wingate test on a wheelchair ergometer in the lab and a Sprint, Illinois, and Spider test in the field, with inertial measurement units on their wheelchairs. Associations were assessed using regression analyses, and construct validity was assessed with an independent *t* test (elite vs. amateur).

**Results:**

The strongest associations were observed between lab outcomes and field sprint power (R^2^ > 90%), followed by peak linear velocity and test duration (R^2^ = 77%–85%), while peak rotational velocity showed the lowest associations with lab outcomes (R^2^ = 69%–80%). The elite group outperformed the amateur group on all test outcomes.

**Conclusions:**

Despite differences in lab- and field-testing methodologies (e.g., trunk influence, linear/rotational components), the strong associations indicate overlap in measured constructs. Field testing offers valuable insight into practical performance, whereas lab testing enables in-depth biomechanical and physiological analyses. All tests effectively discriminate between elite and amateur wheelchair tennis players.

**What Is Known**

- Wheelchair tennis athletes can monitor their performance with both lab and field testing. Lab testing offers detailed biomechanical and physiological measures, while field testing provides higher external validity.

**What Is New**

- The study clarified how lab and field testing complement each other. Strong associations between lab and field sprints suggest that lab-based push-by-push power output can refine players’ propulsion technique in the field. Additionally, strong associations between Wingate and field agility tests reveal players’ strengths and weaknesses, guiding targeted training: players with relatively high anaerobic power should focus on wheelchair mobility, while agile players should work on enhancing anaerobic power.

Wheelchair tennis has been incorporated in the programs of all four Grand Slam tennis tournaments and the Paralympics Games. The only difference from able-bodied tennis is that the ball is allowed to bounce twice. In wheelchair tennis, the wheelchair-athlete combination needs to maneuver on court with a combination of linear velocities, accelerations and rotations.^1^ The anaerobic energy system is assumed to be crucial for this short-term work and wheelchair skills are required for high velocities and rotations.^2,3^ To gain insights into their performance, wheelchair tennis players and their coaches can monitor these characteristics through testing, either in a standardized laboratory or natural field environment.^4–6^

Lab testing provides researchers with standardized conditions to collect detailed physiological, kinetic, or kinematic data.^7^ Common methods for assessing wheelchair sprint and anaerobic power in the lab are Sprint and Wingate tests, conducted on a computer-controlled wheelchair ergometer.^8^ Such a wheelchair ergometer accommodates the player’s sports wheelchair and objectively measures mechanical power output.^9^ To achieve velocities similar to those experienced on court, the Sprint test is performed against a resistance that aims to mimic realistic conditions. In contrast, a Wingate test is performed against a higher resistance, which decreases rim velocity. This lower velocity is limiting upper-body coordination difficulties and allows higher power production.^10^

Field testing is conducted on-court, and this is easier to perform and organize by the player/coach and likely enhances external validity by reflecting real-world conditions.^6^ Wheelchair mobility performance, that is, the player’s ability to maneuver on-court, can be systematically evaluated using tests such as the 10 m straight Sprint test, the Illinois agility test, and the Spider maneuverability test.^5^ Attaching inertial measurement units (IMUs) to the wheelchair allows for the assessment of dynamic aspects like linear and rotational accelerations and velocities.^11^ Recent work by van Dijk et al. (2023) showed that by adding a single IMU on the trunk to the existing IMU setup, power output per push during straight-line sprinting can be determined.^12,13^ The Sprint test gives insight in linear velocities and power output, the Illinois test assesses linear and rotational velocities, and the Spider test focuses on rotational velocities. These field tests are construct-valid, meaning that they can distinguish between elite and youth athlete performances.^5^

Lab and field tests are considered complementary, each with unique strengths. However, their similarities and differences are not fully understood, limiting how they should be integrated. Both tests overlap in measuring anaerobic power due to their short duration (<40 seconds) and use of the player’s own sports wheelchair.^2,14^ Except for the Wingate test, all tests are performed with a racket and high (linear or rotational) velocities are achieved that require a certain skill level. Differences include variable rolling resistance in the field due to the athlete’s weight shifting between the smaller front wheels and the larger rear wheels, with the smaller wheels experiencing more resistance.^15^ Conversely, in a lab setting, the large rear wheelchair wheels are strapped onto the rollers of the wheelchair ergometer, resulting in a more constant rolling resistance. Furthermore, when fixed on the wheelchair ergometer, the influence of the player’s trunk is constrained, rotational aspects cannot be assessed and there is no need for small steering adjustments to prevent or correct directional errors. Conversely, in the field, trunk motion has a greater impact, rotational movements are involved, and immediate self-correction is essential to address directional errors.^15,16^

These similarities and differences lead to the question how we should use both testing environments together. To illustrate, if the lab and field sprints are similar, detailed power output analyses in the lab can directly be used for on-court training, for example, an inefficient stroke pattern with high negative and peak powers can be distilled from lab testing and should be a focus point for training.^17^ However, if these sprints are not similar, then the lab results may not accurately translate to on-court performance, and training strategies should be adjusted to reflect the specific demands of the field conditions. To our knowledge, only one study associated the lab and field performance, more specifically between a wheelchair-specific Wingate test with time needed to perform a 20-meter Sprint and an agility test, and found good associations in wheelchair basketball players.^18^

The current study aims to examine associations between wheelchair sprint and anaerobic power, assessed in a standardized lab environment, and wheelchair mobility performance, assessed in the field, among experienced wheelchair tennis players. We hypothesize that strong associations will be found between the power output and velocity of lab tests (Sprint and Wingate) and the power output, velocity, and test duration of the field Sprint test. Additionally, we expect moderate associations between the power output and velocity of lab tests (Sprint and Wingate) and the peak linear velocity, peak rotational velocity, and test duration of the Illinois and the Spider test. Furthermore, this study provides an initial assessment of construct validity by comparing an elite and amateur adult wheelchair tennis group. Given the performance differences between these groups, it is expected that elite athletes will outperform amateurs in both field and lab tests. In line with Rietveld et al. (2019), who assessed these factors in the used field tests but not in lab tests, elite athletes are expected to achieve higher power output, along with greater linear and rotational velocities in the field, leading to faster completion times.^5^ In the lab, elite athletes are expected to achieve higher power output and velocities, compared to amateur players.

## MATERIAL AND METHODS

### Participants

Elite and amateur wheelchair tennis players were included in this cross-sectional study. International and talented wheelchair tennis players (selected by the Royal Dutch Lawn Tennis Association (KNLTB, i.e., dutch abbreviation)) were identified as elite players, players at regional clubs were identified as amateur players. Inclusion criteria were to practise wheelchair tennis on a regular basis (at least one time a week) and the absence of medical contraindications according to the Physical Activity Readiness Questionnaire (PAR-Q, ACSM: 2009). The local ethics committee of the Centre for Human Movement Sciences, University Medical Centre Groningen, University of Groningen, the Netherlands, approved the study protocol (202000455). All participants gave their written consent before participation.

### Measurement Set-up

Lab measurements were performed in two different laboratories with similar facilities (University Medical Centre Groningen and Reade Rehabilitation Centre Amsterdam). Field tests were performed at different training facilities (National Tennis Centre Amstelveen, Drachtster Lawn Tennis Club Drachten and Tennis and Squashclub Haren) on similar acrylic hardcourt surfaces. There was a minimum of 2 days and a maximum of 2 weeks between lab and field measurements. Players used their own sports wheelchair and racket for both tests, with wheelchair tires inflated to the recommended pressure beforehand.

### Standardized Lab Testing

**FIGURE 1:**
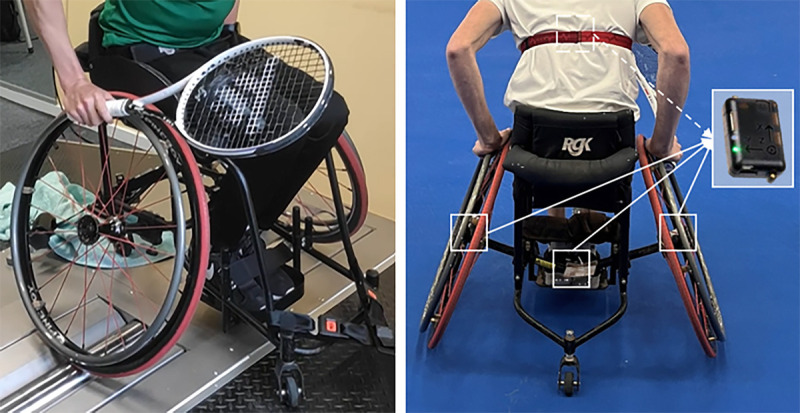
Left: wheelchair tennis player in sports wheelchair on Esseda wheelchair ergometer. Right: placement of three inertial measurement units on wheelchair and a 4^th^ on the player’s chest.

The computer-controlled Esseda wheelchair roller ergometer was used for testing (Fig. 1, Lode BV, Groningen, the Netherlands). This commercial dual-roller wheelchair ergometer provides an accurate individual simulation of inertia and resistance, while allowing for accurate measurements (100 Hz) of both left and right-hand propulsion characteristics.^9^ Before each test, the individual-wheelchair combination was calibrated on the ergometer to account for static and dynamic friction.^9^

### On-court Field Testing

Inertial Measurement Units (IMU) (NGIMU, x-io technologies, Bristol, UK) were placed on the hub of both wheels, the frame of the wheelchair and the chest of the participant (Fig. 1).^11^ The three wheelchair IMUs allow calculation of covered distance, and linear and rotational velocities over time. The chest IMU determines trunk orientation in relation to the global earth frame, enhancing power output accuracy.^16^ All data were collected at 200 Hz via Wi-Fi, allowing synchronous data collection from all four IMUs.

### Test Protocols

#### Standardized Lab Testing

After a familiarization period of 5 minutes, participants were asked to perform the following three tests on the wheelchair ergometer: 1) an isometric strength test without racket, 2) a 10-sec Sprint test with racket, and 3) a 30-sec Wingate test without racket. These protocols are extensively described by Janssen et al. (2022).^4^ In short, regarding the isometric strength test, players were instructed to exert maximum force on the hand rim three times for 5 seconds. The 10-sec Sprint test was performed with a resistance similar to an average gym court (0.012), instead of the slightly lower resistance of an indoor tennis court (0.008), due to the test being part of a standardized battery for various wheelchair sports.^19^ Lastly, the 30-sec Wingate test, also from a standstill, was performed against a high individualized resistance, based on their performance on the isometric strength test.^4^ The isometric strength test was solely used to calculate the Wingate resistances but was not used as outcome measure.

#### On-court Field Testing

**FIGURE 2:**
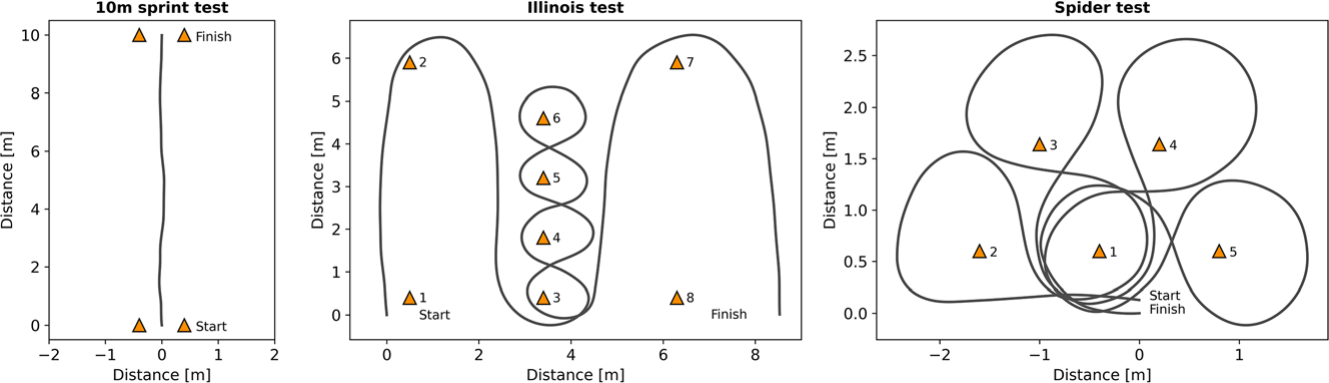
Layout of set up and trajectory of the three field tests.

Participants were asked to complete three field tests: a 10 m Sprint test, an Illinois test, and a Spider test, all with racket (Fig. 2). Each test was performed twice. These tests are specifically developed for wheelchair tennis and extensively described by Rietveld et al. (2019).^5^ Although analyzing the average of the two attempts is considered the most reliable approach for these field tests,^5^ not all players had two good attempts. Consequently, the test with the fastest end time was chosen for further analyses.

To be able to determine power output during the 10 m Sprint test, four coast-down trials were performed to determine drag forces.^20^ Players were instructed to push the wheelchair twice, place their hands on their knees, sit still, and let the wheelchair decelerate naturally for 2 seconds. The first two trials were in an upright position, the last two in a forward bending position. This was performed to evoke a variance in load distribution, such that the rolling resistance coefficients of each pair of wheels could numerically be solved.^21^

### Data Analyses

All analyses were done with custom-written Python scripts, and all used functions can be found in the Worklab and Wheeltennis package.^22,23^

#### Standardized Lab Testing

Torque and velocity data were directly measured from the wheelchair ergometer and filtered using a fourth order low-pass Butterworth filter with a cutoff frequency of 10 Hz. The instantaneous mechanical power output (PO [W]) at each side was calculated from the measured torque (M [Nm]), wheel radius (r_w_ [m]), and wheel velocity (v_w_ [m·sec^−1^]):


$$\mathrm{PO}=M*{r_{w}}^{-1}*v_{w}$$ [equation 1]

Regarding the 10-sec Sprint test, only data of the first 10 m was analyzed for more resemblance with the 10 m field test. Sprint peak velocity (v_peak_ [m·sec^−1^]) was calculated over the average of left and right wheel velocity, as the highest peak sample. Average sprint power output (PO_mean_ [W·kg^−1^]) and Wingate anaerobic power (P30 [W·kg^−1^]) were calculated as the sum of left and right wheel, averaged over 10 m or 30 seconds, respectively, and divided by body mass.

#### On-court Field Testing

Start time for every test was defined as when initial linear velocity exceeded 0.1 m·sec^−1^. End time for the Sprint test was defined as the moment when the average distance covered by both wheels was 10 m. For the Illinois and Spider test, end times were set based on analyses of plots created in Python (Fig. 2). A zero line was set at the starting point; when this line was crossed at the end of the trial, time was automatically defined.^5^

The three-dimensional gyroscopes of the IMUs were used to derive all variables. Data was filtered with a low-pass second order recursive Butterworth filter with a cutoff frequency of 10 Hz.^11^ Because of the camber angle and horizontal rotations of the sports wheelchair, angular wheel velocity was first corrected using the sinus of the camber angle and the gyroscope data of the frame sensor in the Z-direction (equation 2).^5^ Linear velocity (v_lin_ [m·sec^−1^]) was subsequently calculated from angular wheel velocity (v_ang_ [°·sec^−1^]) and wheel circumference (WC [m]) (equation 3). Rotational velocity (v_rot_ [°·sec^−1^]) was directly obtained from the GyroZframe.^24^


$$v_{\mathrm{ang}}=\left({\text{GyroY}}_{\text{wheel}}+\sin\left(\text{camber}\right)\right)*{\text{GyroZ}}_{\text{frame}}$$ [equation 2]

$$v_{\mathrm{lin}}=\mathrm{WC}/360^{{}^{\circ}}*v_{\mathrm{ang}}\;$$ [equation 3]

As the wheelchair is assumed to have no angular rotations during the sprint, air resistance is assumed negligible, and the floor is assumed flat, cycle-average mechanical power (PO [W]) was determined by summing the power loss due to rolling resistance (F_roll_ [N]) and the change in kinetic energy (equation 4). F_roll_ was determined from the coast-down tests and corrected for trunk inclination to obtain realistic rolling resistance forces.^16,21^ The change in kinetic energy was determined from wheelchair velocity (v_lin_ [m·sec^−1^]) and total mass of wheelchair and user (m_total_ [kg]).^12^ Cycle time is represented by T [sec]. To obtain average power output, the weighted average of power output per cycle was calculated.


$$\mathrm{PO}=\left(1/T\right)*\int_{0}^{T}\bar{-F_{\text{roll}}*v_{\mathrm{lin}}+\frac{1}{\mathrm{dt}}\left(0.5*m_{\text{total}}*{v_{\mathrm{lin}}}^{2}\right)}\;$$ [equation 4]

Mean power output (PO_mean_ [W]) was only calculated for the Sprint test. Peak linear velocity (v_peak-lin_ [m·sec^−1^]) was calculated for the Sprint and Illinois test and peak rotational velocity (v_peak-rot_ [°·sec^−1^]) for the Illinois and Spider test. Test duration [sec] was an outcome measure for all three field tests.

### Statistical Analyses

Statistical analyses were done using Python 3.8 (Python Software Foundation). Average and standard deviation for the outcomes were calculated, separately for elite and amateur players. To check for systematic differences between elite and amateur players (e.g., construct validity), an independent *t* test with Bonferroni correction was performed. This implies that the chosen alpha level (0.05) was divided by the amount of *t* tests (i.e., 11). Significance level for the *t* test was thus set at 0.05/11 = 0.0045.

To assess the associations between lab and field test outcomes, linear or curvilinear regression analyses were performed. The regression (linear or curvilinear) with the highest explained variance (*R*^2^) was reported. Lastly, because mean power output and peak linear velocity were identical variables for lab and field sprints, a dependent *t* test was used to check for systematic differences.

## RESULTS

**FIGURE 3:**
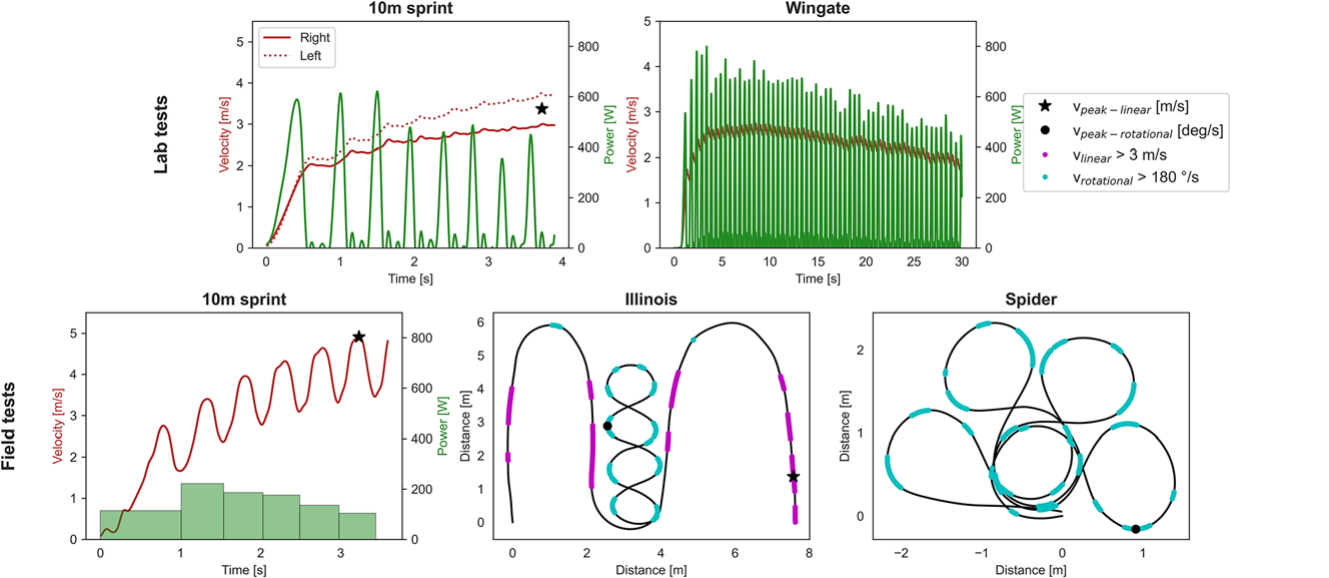
Typical example of outcomes from lab (top row) and field tests (row below). Velocity is shown in red, power output in green and completed trajectory in black. Peak linear and rotational velocity is annotated and the Illinois and Spider test are highlighted when linear velocity is above 3 m·sec−1 (purple) or rotational velocity is above 180 °·sec^−1^ (light blue).

The KLNTB identified nine players as elite and an equal amount of amateur players were included, resulting in a total of 18 wheelchair tennis players (Table 1). All participants completed all lab and field tests. Because of technical issues of the wheelchair ergometer two participants had missing data for the 10-sec Sprint lab tests, and one participant had missing data for the Wingate test. Power output of the 10 m field Sprint test was missing for six players. Three elite players did not perform the coast-down tests, two amateur players had pushes in their coast-down tests, and for one amateur player, the data file was corrupted. Table 2 reports the main test outcomes and typical examples of the lab and field tests results are displayed in Figure 3. The independent *t* test showed that the elite group performed consistently better on all tests, compared to the amateur group (Table 2).

**TABLE 1:** Participant characteristics

	*N* or Mean (SD)	
Participant Characteristics	Elite Group	Amateur Group
Men/women (*n*)	*5/4*	*5/4*
Age (years)	23 (7)	42 (18)
Body mass (kg)	61 (7)	81 (21)
Wheelchair tennis experience (years)	10 (8)	13 (13)

**TABLE 2:** Primary test outcomes for the Sprint and Wingate tests in the lab, and for the Sprint, Illinois and Spider tests in the field for the elite and amateur wheelchair tennis players

		Amateur Group		Elite Group				
		*N*	Mean (SD)	*N*	Mean (SD)	*df*	t Statistic	*P*
**Lab tests**								
**Sprint test**								
PO_mean_ [W·kg^−1^]	Average power over 10 m	*8*	1.1 (0.5)	*8*	2.2 (0.6)*	14	4.25	<0.01
v_peak_ [m·sec^−1^]	Peak velocity over 10 m	*8*	2.6 (0.4)	*8*	3.5 (0.4)*	14	4.33	<0.01
**Wingate test**								
P30 [W·kg^−1^]	Average power over 30 seconds	*8*	1.2 (0.5)	*9*	2.5 (0.4)*	15	6.62	<0.01
**Field tests**								
**10 m Sprint test**								
PO_mean_ [W·kg^−1^]	Average power output	*6*	0.8 (0.3)	*6*	2.3 (0.4)*	10	7.99	<0.01
v_peak-lin_ [m·sec^−1^]	Peak linear velocity	*9*	3.2 (0.6)	*9*	4.6 (0.4)*	16	5.52	<0.01
Test duration [sec]	Total time needed	*9*	4.9 (0.6)	*9*	3.5 (0.2)*	16	−6.44	<0.01
**Illinois test**								
v_peak-lin_ [m·sec^−1^]	Peak linear velocity	*9*	2.8 (0.4)	*9*	4.0 (0.4)*	16	6.51	<0.01
v_peak-rot_ [°·sec^−1^]	Peak rotational velocity	*9*	173 (17)	*9*	240 (21)*	16	7.23	<0.01
Test duration [sec]	Total time needed	*9*	31.3 (6.7)	*9*	18.9 (1.3)*	16	−5.48	<0.01
**Spider test**								
v_peak-rot_ [°·sec^−1^]	Peak rotational velocity	*9*	176 (24)	*9*	260 (23)*	16	7.44	<0.01
Test duration [sec]	Total time needed	*9*	24.4 (3.8)	*9*	15.6 (1.2)*	16	−6.66	<0.01

*Indicating a significant difference between amateur and elite players.

### Associations Among Lab and Field Tests

All associations were determined with 17 or 16 individual data points, except for the power output of the field sprint with the lab tests; those three regressions were constructed with only 11 individuals.

**FIGURE 4:**
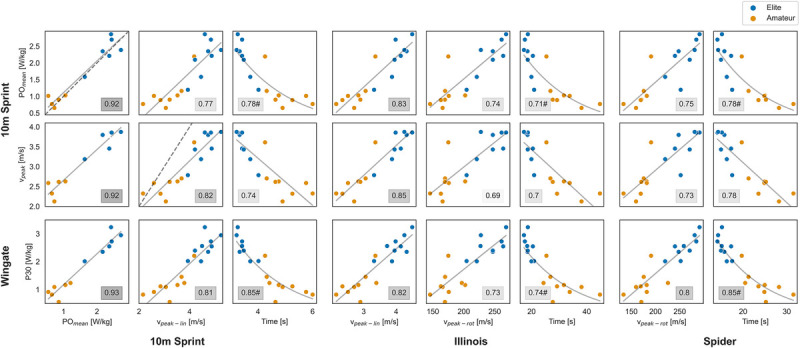
Association between lab and field test outcomes. Lab tests are displayed at the vertical axis, field tests at the horizontal axis. Plots are annotated with the explained variance (R^2^), if the curvilinear regression gave the best fit, an # is added to the R^2^. Two plots show the line of identity (dotted line) because PO_mean_ and v_peak_ were measured in both lab and field sprints. Orange dots designated the amateur players and blue dots are elite players.

The strongest associations (R^2^ > 90%) were found for the power output in the field Sprint test with all three lab outcomes. Peak linear velocity and time needed for all field tests showed slightly lower explained variances (R^2^ = 77%–85%) with the three lab outcomes. Peak rotational velocity showed the lowest explained variances (R^2^ = 69%–80%) with lab outcomes. Figure 4 shows the associations between the lab and field test outcomes.

Peak linear velocity and average power output were both measured in the lab and field sprints and a line of identity is shown in these two plots in Figure 4 (dotted line). Peak linear velocity was on average significantly 26 ± 14% higher in the field sprint, that is, t(15) = 7.39, *P* < 0.001. Mean power output was not significantly different in the field sprint, compared to the sprint in the lab, that is, t(10) = −1.07, *P* = 0.31.

## DISCUSSION

The associations between wheelchair sprint and anaerobic power, assessed within a lab setting, and wheelchair mobility performance, evaluated on the field, are strong for experienced wheelchair tennis players. As expected, the field Sprint test outcomes showed the strongest associations with the lab Sprint and Wingate test outcomes. However, and in contrast with our hypothesis, Illinois and Spider test outcomes, that mainly focused on rotational velocities and require different skills, also showed strong associations with the lab tests outcomes. Thus, although there are some differences in set up between lab and field testing (i.e., influence of trunk, linear or rotational components, skill involvement), the strong associations show that these tests partly measure the same construct (i.e., sprint performance, anaerobic power and wheelchair mobility). Furthermore, the expected construct validity of both lab and field tests was confirmed, as all tests outcomes differentiated between elite and amateur players.

The current study found strong associations between results of the wheelchair-specific lab and field test battery. To our knowledge, only one study reported wheelchair-specific associations between a Wingate test and field sprint test among wheelchair basketball players and found comparable associations (R^2^ = 86% in their study, compared to 85% in this study).^18^ No wheelchair-specific studies investigated the relation with agility tests, like the Illinois and Spider test, but the strong associations in the current study showed that there is overlap in test requirements. In contrast, studies using an arm-crank ergometer for the Wingate test exhibited weaker associations with linear field wheelchair sprints (R^2^ = 48%) and wheelchair agility tests (R^2^ = 32%).^25,26^ The stronger associations between wheelchair-specific testing modalities highlights the need for wheelchair-specific lab testing methodologies.

Both lab and field tests showed initial evidence of construct validity, as they were able to distinguish between amateur and elite players. This validation aligns with findings reported by Rietveld et al. (2019) on wheelchair tennis field tests and extends it to lab testing.^5^ Although groups differed significantly from each other, regression analyses were conducted on the entire group of players without playing level as confounder, due to the small sample size. To examine the influence of playing level on the associations, a larger sample size should be included. The curvilinear relations in Figure 4 already suggest an influence of playing level. Curvilinear associations were found between test duration of the 10 m field Sprint, Illinois and Spider tests with the lab Sprint and Wingate test, indicating that amateurs perform relatively better in the lab but require more time for the field tests. The higher skill level of elite players becomes more apparent in field tests. After making an error when pushing the hand rim, immediate correction is needed in the field, whereas these adjustments are not necessarily required on the wheelchair ergometer.

Graphs for sprint velocity between lab and field differ in amount of deceleration after every push (Fig. 3). In the lab, the wheelchair is fixed to the wheelchair ergometer and allows no movement in relation with the trunk. Conversely, in the field, the wheelchair counteracts with the movement of the trunk and thus exaggerates the actual velocity of the total wheelchair-user combination, both in a positive and negative direction. Moreover, rolling resistance is assumed close to constant on the wheelchair ergometer, but fluctuating in the field because the mass of the user shifts between the smaller front castor-wheels (that have a larger rolling resistance) and the larger rear wheels. Not only deceleration after every push differed, peak velocity was also significantly higher in the field (26 ± 14%). This can at least partly be explained by the lower resistance coefficient of 0.008 on hard-court, compared to 0.012 used on the wheelchair ergometer.^19^ Future studies should use similar resistances to better isolate and investigate the impact of trunk motion.

In contrast to velocity, mean power output did not differ significantly between lab and field sprints. Power output also showed a strong association between lab and field (R^2^ = 92%). Caution is warranted in the interpretation of the association due to the limited power output data in the field (*N* = 11) and clustering among players in the scatter plot, that is, amateur players are in the lower left corner, elite players in the upper right corner (Fig. 4). The analyzed players exhibited values closely aligned with the line of identity (dotted line in Fig. 4) and did not show a significant difference in POmean (*P* = 0.31). Therefore, it might be plausible that any missing values in the current study could also fall near this line of identity, potentially preserving the strong association. Wheelchair tennis players may encounter varying levels of rolling resistance, arising from different surfaces or slight disparities in tire pressure, that will influence the achieved velocity.^19^ In contrast, while power output takes rolling resistance and velocity into account, it emerges as a more objective measure to utilize.

### Practical Implications

Strong associations suggest that detailed biomechanical analyses from lab testing can guide training to improve player’s wheelchair mobility on-court. Lab testing offers unique advantages, including detailed power output, independent left-right measurements, and its suitability for tests against higher resistances. Detailed power output can, for example, be used to analyze negative and peak power output during wheelchair propulsion with a tennis racket, which has led already to the development of a new, more efficient hand rim for wheelchair tennis.^17^ The lab setting, without the need for immediate corrections due to directional errors, provides players with more insights into their maximal capacities for the left and right arm separately.^27^ Lastly, higher resistance tests in the lab can be used to measure anaerobic power and to construct force-velocity curves, informing whether players should prioritize training on force or velocity.^3^

Where lab tests can give a deeper understanding of the biomechanics behind a sprint, field tests show the wheelchair mobility on-court, including linear and rotational aspects. An earlier study distracted six key mobility components during wheelchair tennis matches and found that four of them were based on rotations, that can only be tested in the field.^1^ Moreover, field tests are more similar to match situations, as it is performed on the same court as matches, resulting in a higher ecological validity. Lastly, field testing demands immediate correction when deviating from the desired trajectory and requires thus more skill compared to lab testing.

Furthermore, combining both testing environments will give a complete overview of the players’ performance and it can also provide direction for training. Lab and field tests outcomes showed strong associations, but there also remains an unexplained variance (1 − R^2^), due to test-retest variation and to the different focus of the tests.^28^ Because elite players are often seeking for the last few percentages to improve their performance, they will benefit from both lab and field testing, while for amateur players, field tests may also be sufficient to track performance. Additionally, looking at the constructed regression lines, those to the right of the ‘rotational velocity-anaerobic power trend line’ excel in rotational skills, suggesting power-focused training, whereas those with higher power but less rotational skills could benefit more from rotational (skill) training.

To track wheelchair performance, it is recommended to adopt the field test battery, which is easier to perform, regularly throughout the season (e.g., four times a year). Lab testing should complement this regimen for more in-depth biomechanical analyses. Because of the strong associations between lab and field outcomes and the increased effort required for lab visits, the frequency of lab testing may be lower, such as twice a year. Lab tests are ideally performed around the same time as the field tests.

### Future Research

This study focused on standardized wheelchair testing but the actual performance takes place during competition, demanding a holistic integration of skills such as sprinting, rotating, racket handling, and tactical decisions.^29^ The next step is to assess how much these lab and field tests contribute to match performance. Additionally, current methods for determining power output are established for straight-line sprints. As turning involves unknown factors like rotational inertia and increased rolling resistance due to, because of, slipping, the accuracy of IMU-based power estimation during turning is uncertain.^30^ Further developments are necessary to extend power monitoring to turning, enabling objective assessments in tests, training sessions, and competitions for a comprehensive understanding of the players’ workload throughout their season.

## CONCLUSIONS

Among experienced wheelchair tennis players, there are strong associations between wheelchair lab sprint and anaerobic power with wheelchair mobility field performance. Although there are some differences between lab- and field-testing methodologies (i.e., influence of trunk motion, linear and rotational velocity profile, skill involvement), the strong associations indicate overlapping measured constructs (i.e., sprint performance, anaerobic power and wheelchair mobility). This implies that field testing provides us with valuable wheelchair mobility insights, and lab testing gives a broad additional array of in-depth biomechanical and physiological analyses. All test outcomes successfully differentiated between elite and amateur players, providing initial evidence for the construct validity of both the lab and field tests.
